# Primary Squamous Carcinoma: A Description of a Rare Histological Type of Breast Cancer

**DOI:** 10.7759/cureus.69748

**Published:** 2024-09-19

**Authors:** Mohamed Kaakoua, Mohamed Amine Haouane, Soukayna Boujmadi, Moussa Abdoul Aziz Sawadogo, Mohamed Amine Azami, Rhizlane Belbaraka, Ismail Essadi

**Affiliations:** 1 Department of Medical Oncology, Ibn Sina Military Hospital, Marrakesh, MAR; 2 Department of Pathology, Caddi Ayyad University of Marrakesh/Ibn Sina Military Hospital, Marrakesh, MAR; 3 Department of Medical Oncology, Mohammed Vi University Hospital Center, Marrakesh, MAR

**Keywords:** breast cancer, histology, prognosis, squamous cells carcinoma, treatment

## Abstract

Primary squamous cell carcinoma is a very rare histological entity of breast cancer. The prognosis is generally poor, and treatment is mainly based on surgery, associated with systemic therapy and radiotherapy adapted to the profile and stage of the tumor.

We report a case of a 50-year-old woman presented with a right breast lump. Breast ultrasound combined with mammography revealed opacity highly suspicious of malignancy. Breast lump biopsy confirmed the presence of invasive squamous cell carcinoma. The patient was treated optimally with mastectomy and lymphadenectomy, adjuvant chemotherapy, and radiation.

Primary squamous cell carcinoma of the breast remains a rare cancer for which prognosis and treatment are unclear.

## Introduction

Squamous cell carcinoma of the breast is one of the rarest histopathological entities, making up less than 0.2% of all cases of breast cancers [1]. Theoretically, such a tumor develops from squamous metaplasia of ductal carcinoma cells [2]. Diagnosis depends on histology, showing a majority of squamous cell type with no other sources, especially cutaneous ones [2,3]. It is characterized by rapid growth and aggressiveness because of its relative resistance to chemotherapy. This type of cancer, being a rare form, is usually underrepresented in various clinical trials; therefore, the treatment for such patients is not well elaborated.

We report observations from a patient with this tumor and will discuss clinical, histological and prognostic aspects of this rare entity.

## Case presentation

We report the case of a 50-year-old woman with no apparent medical history who was five years postmenopausal and presented with a mass in her right breast, evolving for more than six months. Clinical examination found a 3 cm mass in the junction of the upper quadrants of the right breast mobile in both planes, without inflammatory signs or nipple discharge and without ipsilateral axillary lymphadenopathy.

**Figure 1:**
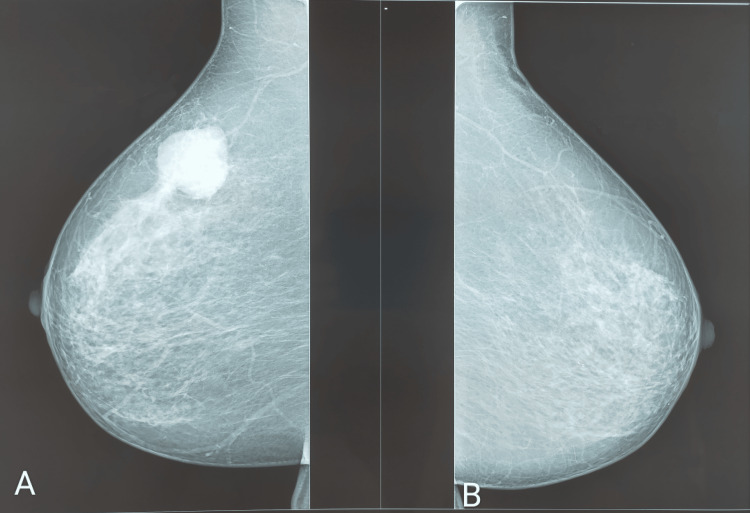
Mammographic appearance of a lesion on the right breast cancer at the junction of the upper quadrant, oblique incidence (35 mm x 20 mm). (A) Right side and (B) left side.

**Figure 2:**
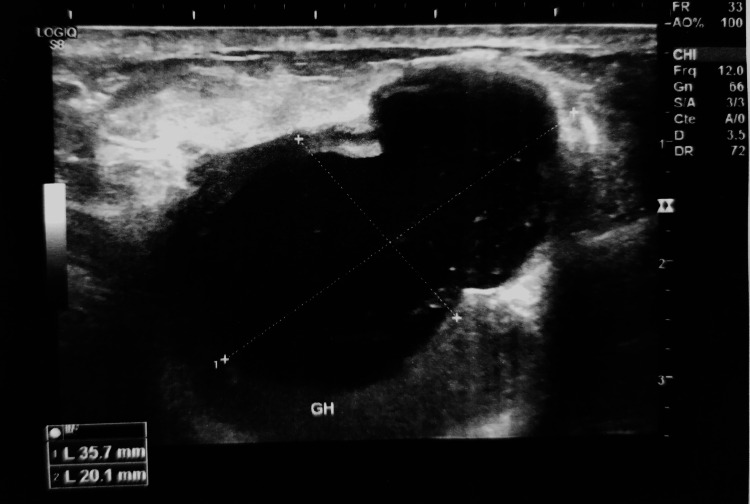
Sonographic appearance of the tumor. Hypoechoic lesion in the junction of upper quadrant with irregular contours measuring 35 mm x 20 mm.

Breast ultrasound coupled with mammography revealed an opacity at the junction of the upper quadrants of the right breast measuring 35 mm x 20 mm with irregular and speculated contour, classified BIRADS 5 (Figures 1-2).

**Figure 3:**
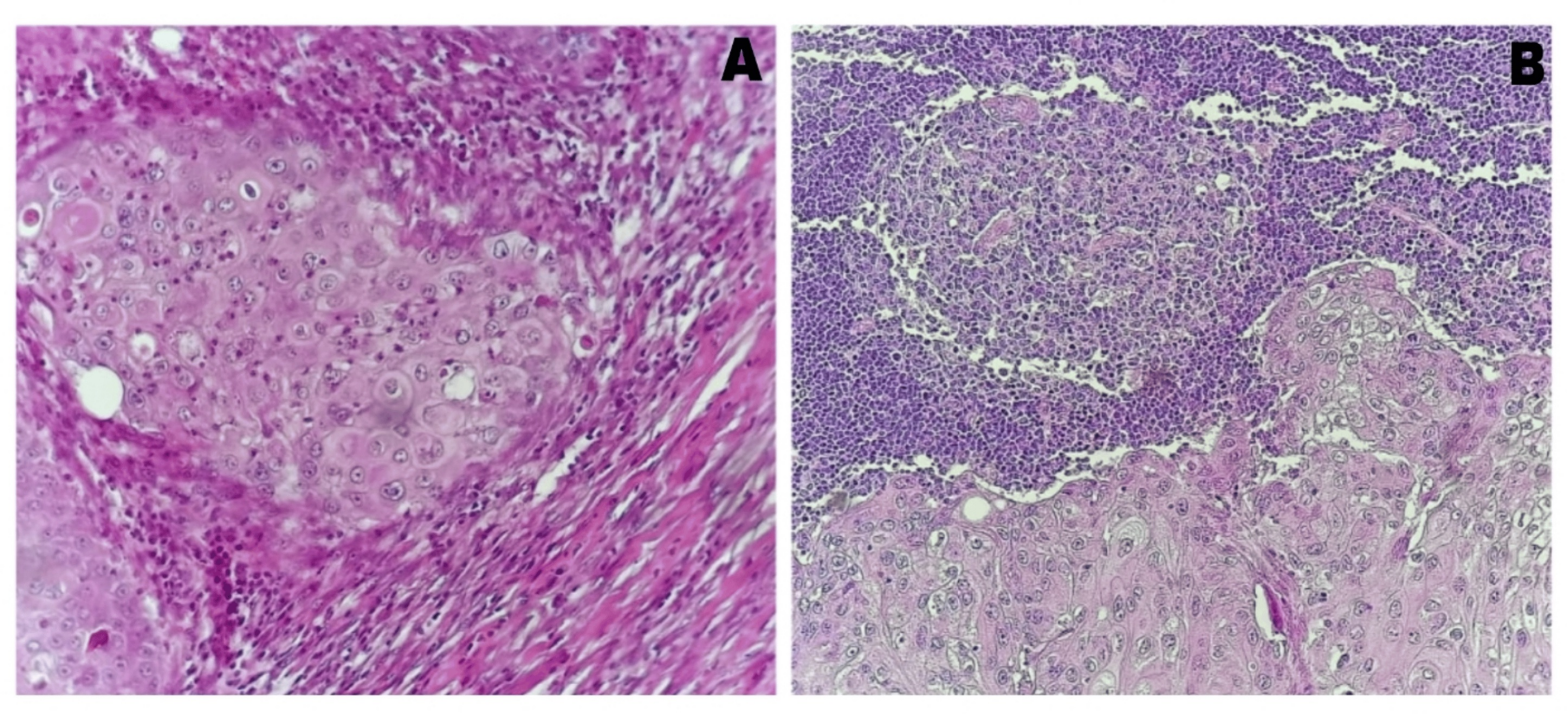
Histological appearance of the breast and lymph node lesions. (A) Squamous cell carcinoma, moderately differentiated: Tumor proliferation shows squamous differentiation (intercellular bridges, focal keratinization); there is mild nuclear pleomorphism, and mitotic figures are frequently observed (hematoxylin-eosin stain, x200). (B) Lymphoid parenchyma invaded by epithelial tumor proliferation showing squamous differentiation consistent with lymph node metastasis of squamous cell carcinoma (hematoxylin-eosin stain, x100).

Breast mass biopsy confirmed the presence of invasive squamous cell metaplastic breast carcinoma. Tumor was SBR grade 3; hormone and HER2 receptor negative, cytokeratin 5/6 positive, and Ki67 at 50%. Chest, abdomen, and pelvic CT scans and bone scans were normal. Clinically, the tumor was classified as cT2N0M0. The patient underwent modified radical mastectomy (Patey's operation) and axillary lymphadenectomy. Definitive histology confirmed a diagnosis of metaplastic infiltrating squamous cell carcinoma without in situ components. The tumor measured 36 mm and exhibited vascular embolism and axillary lymph node involvement (two positive nodes in eight specimens) (Figure 3). The resection margins were clear.

The disease is classified as stage IIb (pT2N1M0). After the decision of the multidisciplinary consultation meeting, the patient received adjuvant chemotherapy (04 cycles of anthracyclines-cyclophosphamide according to the dense dose schedule followed by 12 weeks of paclitaxel) followed by radiotherapy (normofractionated radiotherapy of the chest wall and lymph node areas: internal mammary chain and level II to IV); with a 36-month follow-up without signs of clinical or mammographic recurrence.

## Discussion

Primary squamous cell carcinoma of the breast is a part of a diverse group of metaplastic breast cancers. It is an extremely rare entity whose prevalence does not exceed 0.2% [1]. The presence of a squamous metaplastic lesion without mesenchymal and ductal components histologically defined this tumor, after ruling out other origins of squamous cell carcinoma, such as a pure cutaneous lesion [2].

Primary squamous cell carcinoma of the breast typically occurs in postmenopausal women, with a median onset age of 55 years [3,4]. In our reported case, the patient was 50 years old. According to several reported series, the most frequently observed mode of revelation is the appearance of a breast mass of variable size, rapid evolution, mastodynia, nipple discharge or retraction, and rarely a breast abscess [5,6,7,8]. The radiological appearance is often nonspecific and can be misleading, potentially resembling a benign lesion. Confirmation of the diagnosis of primary squamous cell carcinoma of the breast requires clinical examination and biopsy with pathological study to rule out a skin origin or a secondary location. Immunohistochemically, the tumor frequently expresses the cytokeratins CK14, CK5/6, and CK17 [9]. This is typically a triple-negative phenotype. Gao et al. reported a 14% overexpression of the HER2 receptor in a large series of 47 patients with metaplastic squamous cell carcinoma [10]. Our patient had triple-negative breast cancer. According to earlier studies, lymph node involvement was observed in 10% to 30% of cases [1,11].

Adjuvant therapy for squamous cell carcinoma of the breast stays similar to conventional breast cancer due to a lack of data and clinical studies. However, some authors have reported the benefits of platinum-based chemotherapy compared with traditional breast cancer chemotherapy [12]. In a recent study, a combination of immunotherapy and chemotherapy was tested in patients with advanced metaplastic cancer [13]. The combination showed excellent responses. Further studies testing immunotherapy in this population are expected in localized and metastatic stages [13]. The prognosis of primary squamous cell carcinoma of the breast remains poor, with an estimated mean five-year survival rate of 50% [12].

## Conclusions

Primary squamous cell carcinoma of the breast is one of the rare tumors among metaplastic carcinomas. Clinical and radiologic manifestations are non-specific. In the localized stage, surgery remains the standard of care. The value of adjuvant chemotherapy and radiotherapy remains unclear. The clinical prognosis remains severe. Its *basal-like* profile raises questions about immunotherapy's place in that entity's treatment.
